# Estimation of a significance threshold for epigenome‐wide association studies

**DOI:** 10.1002/gepi.22086

**Published:** 2017-10-15

**Authors:** Ayden Saffari, Matt J. Silver, Patrizia Zavattari, Loredana Moi, Amedeo Columbano, Emma L. Meaburn, Frank Dudbridge

**Affiliations:** ^1^ Department of Non‐Communicable Disease Epidemiology London School of Hygiene and Tropical Medicine London United Kingdom; ^2^ MRC Unit, The Gambia and MRC International Nutrition Group London School of Hygiene and Tropical Medicine London United Kingdom; ^3^ Department of Psychological Sciences, Birkbeck University of London London United Kingdom; ^4^ Department of Biomedical Sciences University of Cagliari Cagliari Sardinia Italy; ^5^ Department of Health Sciences University of Leicester Leicester United Kingdom

**Keywords:** CpG, DNA methylation, epigenetic epidemiology, EWAS, FWER, GWAS, permutation, resampling, simulation extrapolation

## Abstract

Epigenome‐wide association studies (EWAS) are designed to characterise population‐level epigenetic differences across the genome and link them to disease. Most commonly, they assess DNA‐methylation status at cytosine‐guanine dinucleotide (CpG) sites, using platforms such as the Illumina 450k array that profile a subset of CpGs genome wide. An important challenge in the context of EWAS is determining a significance threshold for declaring a CpG site as differentially methylated, taking multiple testing into account. We used a permutation method to estimate a significance threshold specifically for the 450k array and a simulation extrapolation approach to estimate a genome‐wide threshold. These methods were applied to five different EWAS datasets derived from a variety of populations and tissue types. We obtained an estimate of $\alpha =2.4\times 10^{-7}$ for the 450k array, and a genome‐wide estimate of $\alpha =3.6\times 10^{-8}$. We further demonstrate the importance of these results by showing that previously recommended sample sizes for EWAS should be adjusted upwards, requiring samples between ∼10% and ∼20% larger in order to maintain type‐1 errors at the desired level.

## 1 INTRODUCTION

Epigenetic marks are mitotically heritable chemical modifications to DNA and histone proteins, which act in concert to regulate gene expression across developmental stages and tissues (Bird, 2002). The most widely studied of these marks is DNA methylation, describing the addition of a methyl group to the five carbon of cytosine bases to form 5‐methylcytosine (5mC), occurring predominantly in the context of CpG dinucleotides. DNA methylation plays a crucial role in cellular processes such as embryonic development, parental imprinting and X‐inactivation. Aberrant methylation patterns have been associated with a number of diseases (Robertson, 2005), and variation in methylation between individuals could potentially explain a proportion of phenotypic variance (Rakyan & Beck, 2006). These observations in particular have led to the popularisation of epigenome‐wide association studies (EWAS), which profile methylomic variation genome wide in the context of normal development and in disease (Rakyan, Down, Balding, & Beck, 2011).

The growth of EWAS can be at least partially attributed to the introduction of the Illumina Infinium HumanMethylation450k BeadChip (450k array) (Illumina, San Diego, CA, USA). The 450k array is a low‐cost, high‐throughput, platform that interrogates around 450,000 individual CpG sites across the genome, covering <2% of all known CpG sites, and 99% of RefSeq genes (see Dedeurwaerder et al., 2011, for a description of the technology). The platform has been used in investigations into the role of methylomic variation across a range of phenotypes and health conditions including cancers (Heyn et al., 2013; Walter et al., 2012), autoimmune disorders (Liu et al., 2013; Swan, Maxwell, & McKnight, 2015), psychiatric conditions (Feinberg et al., 2015; Song et al., 2014; Walton et al., 2015), age‐related phenotypic changes (Florath, Butterbach, Müller, Bewerunge‐Hudler, & Brenner, 2014) and environmental exposures (Joubert et al., 2014; Laufer et al., 2015; Silver et al., 2015). More recently, Illumina have introduced the Infinium MethylationEPIC BeadChip – which offers approximately double the coverage of the 450k (Moran, Arribas, & Esteller, 2016). Although future studies will adopt the latest platform, the majority of publicly available datasets are from the 450k; such datasets are still being generated, so that 450k data will continue to be analysed for years to come.

For such studies, as was previously the case with genome‐wide association studies (GWAS) of single nucleotide polymorphisms (SNPs), the development of standardised experimental design protocols and statistical methods is crucial for ensuring that reported findings are robust, reproducible, and biologically relevant (Michels et al., 2013; Mill & Heijmans, 2013; Rakyan et al., 2011). Although there are signs that analytical frameworks are beginning to crystallise for EWAS, there is one particular aspect that has not received much attention, and that is the level of evidence required for an identified difference in mean methylation levels of a CpG between experimental conditions (a differentially methylated position, DMP) to reach genome‐wide significance. By contrast, early standardisation of the genome‐wide significance threshold was a key factor in the immediate success of GWAS (Dudbridge & Gusnanto, 2008; Hoggart, Clark, De Iorio, Whittaker, & Balding, 2008; Pe'er, Yelensky, Altshuler, & Daly, 2008).

Establishment of a significance level for EWAS is complicated by the fact that multiple CpG sites are tested for association simultaneously, and that sites in close proximity can have correlated methylation states (co‐methylation) at genomic distances of up to 1–2 kb (Eckhardt et al., 2006; Kuan & Chiang, 2012; Ong & Holbrook, 2014). These problems of multiple testing and dependence have previously been addressed in GWAS studies; however, there is no counterpart in EWAS of the well‐understood phenomenon of linkage disequilibrium (LD) that underpins GWAS. In the case of LD, shared ancestry is responsible for the correlation between SNPs, whereas the precise mechanism generating co‐methylation is unknown. It is entirely possible that LD could itself be generating dependency between CpG sites based on their physical proximity. Indeed, a previous study found that some sets of correlated methylated CpG sites appeared to be associated with SNPs in LD blocks (Liu et al., 2014). The extent of co‐methylation might also depend on functional context (Schildknecht, Olek, & Dickhaus, 2015). For example, sites located in CpG islands (regions of high CpG density, often found in gene promoters) would be expected to display a high degree of co‐methylation, as combined they form a functional unit involved in gene silencing.

The presence of a correlation structure within methylation data has at least two major implications for downstream analysis. First, for single site‐level analysis, dependence between measures should be taken into account when estimating a significance threshold. Second, the existence of correlated blocks of methylated CpGs can alternatively be exploited as a means for grouping together multiple sites into regions. The goal of such an analysis is then to identify differentially methylated regions (DMRs), which might be preferred over an individual site‐level analysis aiming to identify DMPs (Rakyan et al., 2011; Robinson et al., 2014; Jaffe et al., 2012).

Regional analysis has two major limitations that perhaps explain why EWAS results still tend to report differential methylation for individual CpGs. Some of the more popular regional methods are optimised for data generated by specific platforms. For example, the “bump hunting” method of Jaffe et al. (2012) when applied to 450k data covers only around 20% of the CpGs profiled on the array (Ong & Holbrook, 2014). Even in cases where the method is designed for the specific platform in question, there is no guarantee that all CpGs can be assigned to regions. This is because CpG sites are distributed non‐uniformly throughout the genome in regions of both high and low density; therefore, it is likely that a number of sites will not form part of any correlated blocks. Ong and Holbrook (2014) found that their region detection method, though designed for the 450k, does not include 24% of the probes on the array, leading the authors to suggest that single probe analysis should be performed alongside region discovery to maximize discovery of differential methylation signals.

For these reasons, we focus here on a significance threshold for single site analysis. Previously Tsai, Spector, and Bell (2012) suggested that a Bonferroni‐adjusted threshold of $\alpha =10^{-7}$, accounting only for those CpGs tested on the 450k, would be overly conservative, and recommended false discovery rate or permutation methods for controlling the error rate until a consensus is established. Rakyan et al. (2011) proposed a liberal threshold of $\alpha =10^{-6}$, based on a hypothetical set of 500,000 CpG probes with sufficient and uniform spacing between probes such that independence of individual measurements is assumed. However, the authors acknowledged that due to correlation between neighbouring CpG sites, a more formal estimation is required, and a stringent level is more likely to lie between 10^−8^ and 10^−7^.

In the context of GWAS, it was argued that the responsible use of a *P*‐value threshold should allow for all polymorphisms across the genome (Dudbridge & Gusnanto, 2008), not only those tested, an argument which applies equally well to EWAS. Essentially, the prior odds of association for any single CpG depend on the total multiplicity of the methylome, not the number present on a commercial product. The methylome has multiplicity of roughly the same order of magnitude as SNPs in the genome with approximately 28 million CpG sites that could be interrogated, although technologies such as the 450k and EPIC measure far fewer.

Without knowledge of the correlation generating mechanism in EWAS, we apply the simulation extrapolation approach of Dudbridge and Gusnanto (2008) to several real 450k DNA methylation datasets to estimate significance thresholds for a hypothetical array with infinite CpG site density. We consider a variety of study populations and cell types to generate specific significance thresholds before drawing a consensus across the studies.

## 2 MATERIALS

The DNA methylation datasets used here were taken from five existing EWAS which utilised the 450k array, see Table 1. Two of these were recent studies in which some of us have been involved, and the remaining three were taken from publicly available datasets deposited in the NCBI Gene Expression Omnibus (http://www.ncbi.nlm.nih.gov/geo/), accessed and downloaded using the *R* package *marmal‐aid* (http://marmal-aid.org) (Lowe & Rakyan, 2013). These studies were selected as some of the largest publicly available datasets representing different ancestral populations and cell types at the time of carrying out the study.

**Table 1:** Details about the five 450k datasets used

Dataset	GEO accession	Population	Tissue	Age	Status	*N*
Gambian	GSE59592	African‐Gambian	Blood	2–8 months	Healthy	120
CRC	N/A	Caucasian‐European	Colon/rectum	58–80 years	Colorectal cancer	18
Caucasian	GSE40279	Caucasian‐European	Blood	19–101 years	Healthy	426
Afr‐Am‐GTP	GSE72680	African‐American	Blood	13–48 years	Healthy/Depression/Bipolar	422
Cau‐Am	GSE41826	Caucasian‐American	Brain	13–79 years	Healthy	65

For each of the datasets, the matrix of processed Beta values was provided or downloaded. These were used without any further processing in order to assess correlation structure. For permutation testing, these were converted to *M* values, which have previously shown to possess more desirable distributional properties for differential methylation analysis when compared to Beta values (Du et al., 2010). The following formula was used to convert from Beta to *M* (Du et al., 2010):

(1) Mi= log 2Betai1−Betai.

The first dataset, referred to as ‘Gambian’, comes from an investigation into the effect of in utero exposure to aflatoxin B1 on DNA methylation patterns in a mother/child cohort from The Gambia (Hernandez‐Vargas et al., 2015). Genome‐wide methylation profiles were generated from peripheral blood samples for 120 infants between 2 and 8 months of age. The data were provided in the form of a matrix of processed Beta values, which had undergone quality control and normalisation. Full details on the experimental protocol and analysis method can be found elsewhere (Hernandez‐Vargas et al., 2015).

The second dataset, ‘CRC’, is taken from a study characterising methylation patterns in 18 cases of colorectal carcinoma and four control samples of intestinal mucosa from a European Caucasian population (PZ, LM, AC, unpublished work). An Illumina GenomeStudio report was provided, which contained raw probe intensities, this was first processed using the *methylumi* package in *R* (Davis, Du, Bilke, Triche, & Bootwalla, 2012) to perform colour balance adjustment and quantile normalisation, and to generate a matrix of Beta values. Control samples were removed due to the high level of discordance expected between cases and controls – a result of abberant CpG island methylation (Costello et al., 2000) and other large‐scale methylomic alterations expected in the CRC cases (Uhlmann et al., 2003). The resulting dataset contained only the 18 cases.

A further three datasets were identified by searching the *marmal‐aid* database for healthy controls from Caucasian or African populations, and then repeating the same search this time querying the GEO database directly. Two datasets were identified in *marmal‐aid*, the first, ‘Caucasian’, comes from a study into age‐related changes to methylomic state as profiled in peripheral blood samples from 426 Caucasian individuals, spanning a wide range of ages (Hannum et al., 2013) (GEO accession number: GSE40279). The second, ‘Cau‐Am’, is from an unknown study (incomplete annotation in *marmal‐aid*) consisting of 65 Caucasian‐American controls. For all of these *marmal‐aid* datasets, processed Beta values were used which had undergone quantile normalization and imputation of missing probes (see Lowe & Rakyan, 2013, for further details). These data were taken forward for analysis without any further processing. The final set identified on GEO, ‘Afr‐Am‐GTP’, is from a study utilising 422 healthy individuals from the Grady Trauma Project, investigating the effect of lifetime exposure to stress on prevalence of psychiatric disorders in a predominantly African American population (Gillespie et al., 2009) (GEO accession: GSE72680). The processed *Beta* matrix was downloaded and used without further processing.

## 3 METHODS

### 3.1 Correlation structure

To begin with, the correlation structure within each methylation dataset was qualitatively assessed. This was performed first to demonstrate the existence of correlation between adjacent CpGs in the datasets, and second to identify any potential large‐scale differences in correlation structure between the datasets, which might be attributable to the different tissues, ethnicities, or conditions studied.

For each dataset, the following procedure was carried out to determine the level of correlation between adjacent probes. Using the subset of 46K probes mapping to chromosome 1, Pearson's correlation between each of the probe Beta values was calculated. Next, pairwise inter‐probe distances were calculated by taking the absolute differences between the genomic positions of the CpGs (in bp) as given in the 450k manifest (from the *R* package illuminahumanmethylation450k; Triche, 2012). This list of inter‐pair distances was filtered in order to retain only those with distance less than or equal to 10,000 bp. These remaining probe pairs were then divided into approximately 10,000 bins containing around 400 pairs each, and the median pairwise inter‐probe distance of the bin recorded. The mean pairwise correlation of Beta values per bin was also recorded.

### 3.2 Permutation scheme

For each dataset, and for all probes on the array, a permutation scheme was used to generate an empirical null distribution of *t*‐test values from which a per‐CpG significance level α could be derived. The *t*‐test is commonly used in EWAS, as it seems to be robust to the non‐normality of methylation *M* values (Du et al., 2010) and provides greater power than non‐parametric methods.

Further details on the algorithms used for permutation and subsampling are given in the Supplementary material, but briefly: phenotypic labels were randomly assigned to samples, for sample size $n:\left\lfloor n/2\right\rfloor$ were designated as cases and $\left\lceil n/2\right\rceil$ as controls. The labels were then randomly permuted 10,000 times, and for each of these permutations, independent unrelated sample *t* tests were performed for each CpG and the absolute *t* values recorded.

In order to estimate the significance threshold at the density of CpGs present on the 450k array, the maximum *t*‐test statistic scores for each permutation were taken and corresponding *P* values were calculated (under the assumption of equal variance), and α taken as the 5th percentile of the distribution of minimum *P* values.

### 3.3 Effective number of independent tests

The effective number of independent tests is the number *m* required to obtain the observed α using the Bonferroni correction:

(2) m=0.05/α.

This was calculated for each α derived from the permutation data. To test whether the observed data were consistent with there being an effective number of independent tests, a beta distribution was fitted for each set of permutation minimum *P* values. Under these assumptions, the minimum *P* values from the permutation replicates would be expected follow a beta distribution with parameters:

(3) $$\beta (a=1,b=m^{\prime }),$$

corresponding to the Šidák correction:

(4) $$1-{\left(1-\alpha \right)}^{m^{\prime }}.$$

The parameters *a* and *b* of the beta distribution can be estimated using the following method of moments estimators:

(5) $$\hat{a}=\bar{x}\left(\frac{\bar{x}\left(1-\bar{x}\right)}{s^{2}}-1\right),$$

(6) $$\hat{b}=\left(1-\bar{x}\right)\left(\frac{\bar{x}\left(1-\bar{x}\right)}{s^{2}}-1\right),$$

where $\bar{x}$ and *s* ^2^ are the sample mean and variance of the minimum *P* values over the permutations.

Fixing the value of *a* to be 1, the estimate for *b* is:

(7) $$\hat{b}=\frac{1-\bar{x}}{\bar{x}}.$$

Maximum likelihood estimation of $\beta (1,\hat{b})$ was then performed using the *optim* function in *R*, using the methods of moments estimate as the starting point, to obtain a final estimate of $m^{\prime }$, which was compared to the value of *m* obtained from the Bonferroni equation given above.

### 3.4 Estimation of genome‐wide thresholds

For each set of permutation results, a subsampling procedure was used to extrapolate the findings to a theoretical array of infinite density (Dudbridge & Gusnanto, 2008). For each permutation, the *P* values were sampled over a uniform grid of 100 densities from 0 to 1, in increments of 0.01, and the minimum *P* value at each density recorded. This procedure was repeated 100 times, and for each of these 100 replicates, the 5% point at each density across all the permutations was recorded. The mean 5% point for each density across all 100 replicates was then used in subsequent analysis.

At lower densities, it would be expected that the majority of sites are not adjacent to each other, and therefore, overall levels of correlation are likely to be low. In this scenario, increasing density will increase the number of independent tests performed, and according to the Bonferroni law, the 5% point should decrease in a manner inversely proportional to the density. However, for higher densities, the level of correlation will increase and as the coverage approaches saturation, the 5% point is expected to converge to an asymptote, which represents α for the entire genome.

To obtain estimates for the asymptote, the Monod function was fitted to each set of effective numbers of tests *m* across the range of densities (Dudbridge & Gusnanto, 2008). The Monod function was initially devised to model the growth of microorganisms, but finds application in other systems where growth is limited by availability of resources. Here, growth can be thought of as the increase in effective number of tests, which is limited by site density. The equation takes the form:

(8) $$f\left(x;u,k\right)=\frac{ux}{\left(k+x\right)},$$

where *u* is the limit as x→∞, and *k* is the value for *x* for which

(9) f(x)=u/2,

it is also known as the half saturation parameter. This function was fitted to the calculated values of *m* with the parameters estimated by least squares and the genome‐wide α estimated as:

(10) α=0.05/u.

Both the permutation and subsampling methods were implemented as python command‐line tools with support for multi‐processing and memory‐mapping to reduce computation time and memory requirements. These are available for download from the first author's github: https://github.com/asaffa/

### 3.5 Sample size estimation

Tsai and Bell (2015) estimated sample size requirements for case‐control and disease‐discordant MZ twin design EWAS based on power simulations across a range of different sample sizes and effect sizes. The simulations were performed using both *t*‐test and Wilcoxon tests, for nominal (*P* = 0.05) and genome‐wide ($P=1\times 10^{-6}$) significance levels. We re‐calculated the sample sizes required for 80% power using our empirical estimates of genome‐wide α.

To this end, the non‐central *t* distributions giving rise to the estimates were inferred from the reported results (table 2 in Tsai and Bell (2015)), and then used to calculate sample sizes for our estimated significance thresholds. The following equation was used:

(11) $$1-\beta =1-F_{\left(n-1,ncp\right)}\left(t_{\left(1-\alpha /2\right)}\right)+F_{\left(n-1,ncp\right)}\left(-t_{\left(1-\alpha /2\right)}\right),$$

**Table 2:** Permutation results showing the 5th percentile of the minimum *P* values from 10,000 permutations of the datasets, *m* is the effective number of tests that this 5% point represents according to the Bonferroni law, and *b* is the estimated *b* parameter after fitting a beta distribution

Dataset	*n*	α	*m*	*b*
Gambian	120	2.04E−07	245,563	170,286
CRC	18	3.53E−08	1,417,410	161,402
Caucasian	426	2.44E−07	204,586	153,670
Afr‐Am‐GTP	422	2.45E−07	204,046	124,186
Cau‐Am	65	3.59E−07	139,451	70,782

where 1−β = 0.8, $F_{\left(n-1,ncp\right)}$ is the cumulative distribution function of the non‐central *t* distribution with *n*–1 degrees of freedom and non‐centrality parameter ncp, given by:

(12) $$ncp=\sqrt{n}\times \frac{\delta }{\sigma },$$

and $t_{\left(1-\alpha /2\right)}$ the critical value when $\alpha =1\times 10^{-6}$. Treated as an optimization problem, this was solved for σ across the range of sample sizes *n* and mean methylation differences δ. These estimates were then used in another optimization, this time solving to obtain sample sizes for each δ using the empirically derived estimates of α.

## 4 RESULTS

### 4.1 Correlation structure

**Figure 1:**
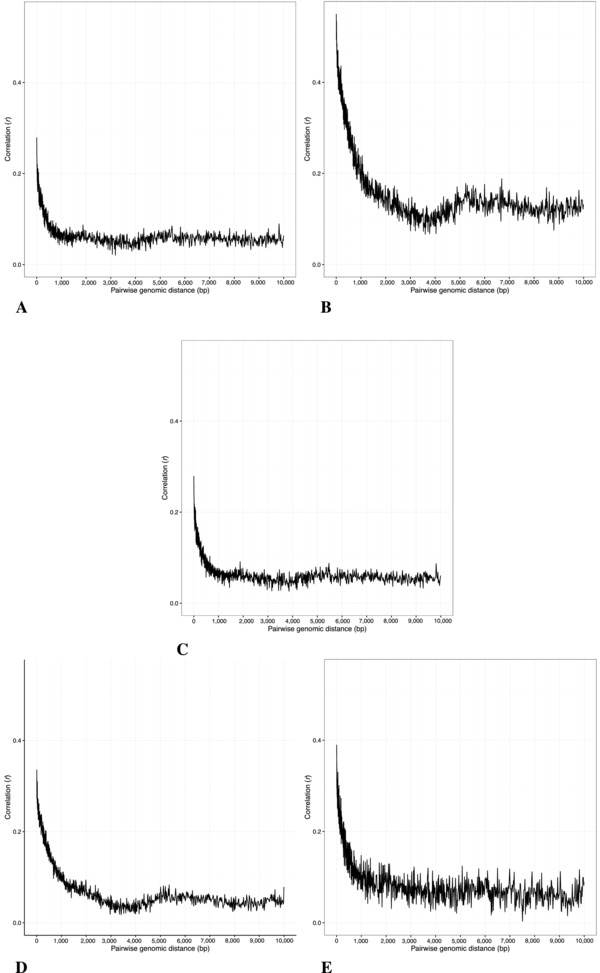
Correlation versus genomic distance for pairs of probes in chromosome 1 *Notes*: (A) Gambian, (B) CRC, (C) Caucasian, (D) Afr‐Am‐GTP, (E) Cau‐Am.

The correlation structure was assessed for each dataset. The plots in Figure 1 show the overall patterns of correlation between methylation status (Beta) of pairs of CpG sites from chromosome 1 as a function of genomic distance. The relationship between these variables appears consistent across datasets. The curves show the same characteristic shape with pairs in close proximity having the highest average levels of correlation, tailing off sharply as distances approach 1 kb, and then decreasing more slowly from 1 to 2 kb, appearing to reach a limit just above zero – assumed to be the mean background level of correlation across the genome. In terms of the actual mean per bin correlation values, these appear comparable across the datasets, with the exception of the CRC set, with the approximate maximum per bin correlations of 0.3, 0.55, 0.4, 0.33, 0.4 and approximate background levels of 0.07, 0.12, 0.04, 0.05, 0.07 for Gambian, CRC, Caucasian, Afr‐Am‐GTP and Cau‐Am sets, respectively. There are also differences in the variability and spread of correlation values between neighbouring bins, perhaps indicating differences in apparent level of noise between sets.

### 4.2 Permutation and effective number of tests

**Figure 2:**
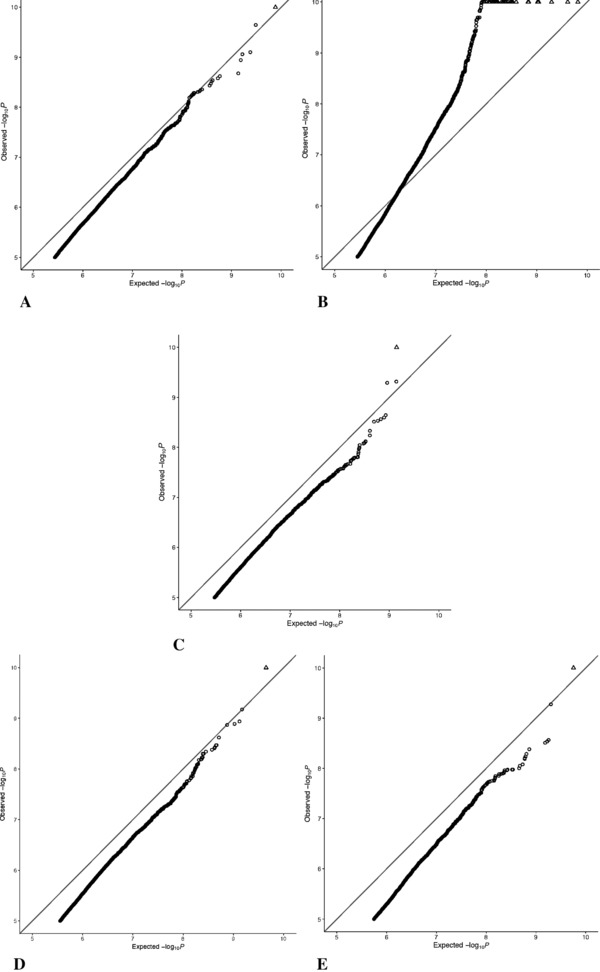
QQ plots showing observed distribution of minimum *P* values verses the expected distribution under complete independence *Notes*: (A) Gambian, (B) CRC, (C) Caucasian, (D) Afr‐Am‐GTP, (E) Cau‐Am.

The results from the permutation testing scheme are given in Table 2. The 5% points of the minimum *p* distributions vary between the different datasets. The αs obtained for Gambia, Caucasian, Afr‐Am‐GTP and Cau‐Am are larger than the Bonferroni adjusted 5% threshold of $\alpha =1.07\times 10^{-7}$, while for CRC, this is smaller by almost a factor of 10. Investigating further, Figure 2 shows QQ plots of the observed minimum *P* distributions against the expected quantiles of the β(1, 467, 624) distribution, which assumes the tests are independent. From these plots, it can be observed for the Gambia, Caucasian, Afr‐Am‐GTP and Cau‐Am sets that while Bonferroni correction produces uniformly distributed *P* values, these are also deflated over the entire range. For the CRC set, it appears that the observed data are not at all well modelled by the beta distribution, and there is not a clear pattern of over or under correction of *P* values, as they appear both deflated at low values and inflated at higher values.

Converting these 5% points to effective number of tests yields results that are not in close agreement to those obtained by fitting the beta distribution. This suggests that the beta distribution with parameters (1, *m*) does not adequately model the minimum *P* value distribution for *m* tests, and thus, while it is a useful modelling tool, there is not a true underlying effective number of independent tests. The results for CRC do not produce the expected distribution of min *P* values, and the estimated values for α, *m* and *b* show little resemblance to the values obtained for the other datasets, indicating potential problems with this particular set. This is perhaps not surprising given that this dataset is very different to the others, not only in disease status and tissue type, but also in having a much smaller sample size. To determine the effect of sample size on the success of the modelling strategy, we repeated the experiment with another small control dataset from an African American population (n=12) from GSE41826 (the same study from which the Cau‐Am set was drawn). This similarly was not well modelled by the beta distribution, also producing an unusually large estimate for the *m* parameter and resulting in α of the same magnitude as that obtained for CRC (see supplementary material).

Assuming the results from the limited analysis of correlation structure performed here are more generalisable, there appear to be only negligible differences between the overall patterns of co‐methylation in the data from the different populations. Therefore, the estimated thresholds for the four groups producing permutation data most closely fitting the expected distribution, Gambian, Caucasian, Afr‐Am‐GTP and Cau‐Am, would seem to be appropriate for combining in order to derive a single figure. Taking the weighted mean of the different estimates for effective number of tests yields a 450k‐specific threshold of: $\alpha =2.4\times 10^{-7}$.

### 4.3 Subsampling and genome‐wide threshold

**Figure 3:**
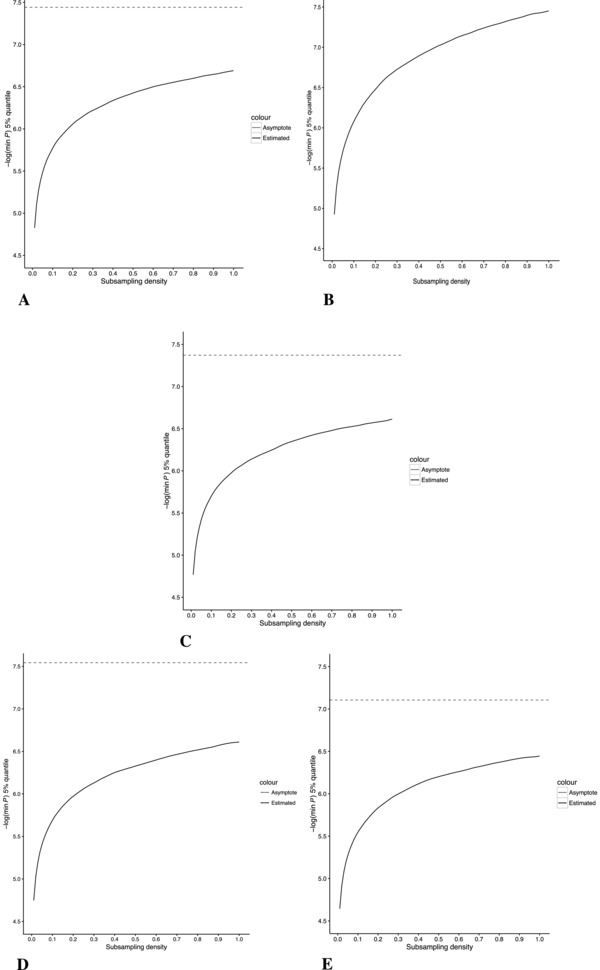
Significance threshold as a function of CpG site density following the subsampling procedure *Notes*: Where possible, the Monod function was fitted to estimate an asymptote representing the threshold at fully saturated CpG density, this is indicated by a dashed line. (A) Gambian, (B) CRC, (C) Caucasian, (D) Afr‐Am‐GTP, (E) Cau‐Am.

**Figure 4:**
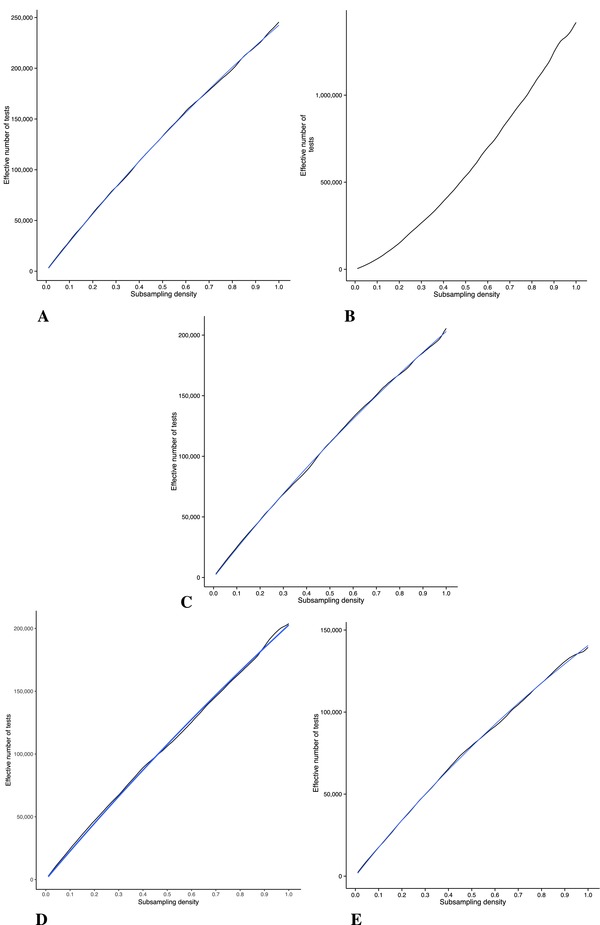
Estimated number of tests as a function of CpG site density *Notes*: Where possible, the Monod function was fitted, this fit is shown by the blue line. (A) Gambian, (B) CRC, (C) Caucasian, (D) Afr‐Am‐GTP, (e) Cau‐Am.

For subsampling, Figure 3 shows the resulting plots of the mean 5th percentile of the sampled minimum *P* values at each subsampling density. The asymptotes were estimated by fitting the Monod function to the effective number of tests, the closeness of the fit can be seen in Figure 4. The resulting estimates for *u*, the limit as the density approaches infinity, and *k*, the value for *x* at which half of genome‐wide multiplicity is accounted for, are given in Table 3. The asymptotes are shown in Figure 3. It was not possible to fit the Monod function to the CRC data. Once again, assuming the results from correlation assessment are more generally applicable, and that there are minimal differences in overall patterns of co‐methylation between the Gambian, Caucasian, Afr‐Am‐GTP and Cau‐Am populations, the weighted mean of the different estimates for the effective number of test can be calculated, giving a genome‐wide $\alpha =3.6\times 10^{-8}$.

**Table 3:** Results from subsampling, showing the final values for the *u* and *k* parameters after fitting the Monod function, and the asymptote representing the genome‐wide significance threshold alpha

Dataset	*u*	*k*	Genome‐wide α
Gambian	1.38E+06	4.71E+00	3.61E−08
CRC	N/A	N/A	N/A
Caucasian	1.18E+06	4.79E+00	4.25E−08
Afr‐Am‐GTP	1.75E+06	7.61E+00	2.86E−08
Cau‐Am	6.38E+05	3.54E+00	7.83E−08

### 4.4 Sample size estimation

Results for the sample size estimations are given in Table 4 alongside the original findings from Tsai & Bell, 2015. Using the 450k‐specific $\alpha =2.4\times 10^{-7}$ increases the sample size estimates by ∼10% over those estimated from power simulations using the suggested threshold of $\alpha =1\times 10^{-6}$. With the estimated genome‐wide $\alpha =3.6\times 10^{-8}$, the sample size estimates are ∼20% larger.

**Table 4:** Sample size estimates based on those presented in Tsai and Bell (2015) (those indicated by an asterisk) using the estimates for 450k and genome‐wide significance derived in this study

	Discordant twin				Case‐control			
Diff	$P<0.05^{*}$	$P<1\times 10^{-6*}$	$P<2.4\times 10^{-7}$	$P<3.6\times 10^{-8}$	$P<0.05^{*}$	$P<1\times 10^{-6*}$	$P<2.4\times 10^{-7}$	$P<3.6\times 10^{-8}$
7	30	178	196	219	37	211	232	259
8	25	145	159	178	30	169	186	208
9	20	117	129	144	24	137	150	168
10	17	98	108	121	20	112	123	138
11	15	81	89	100	17	96	105	118
12	13	71	78	88	15	80	88	98
13	11	63	69	78	13	70	77	86
14	10	55	61	68	11	61	67	75
15	9	50	55	62	10	54	59	66

Diff is the percentage mean methylation difference between case and control, twin and case‐control refer to the study designs, and for each of the significance thresholds,the sample sizes required to achieve a power of 0.8 are given.

## 5 DISCUSSION

In this paper, we first examined the correlation structure in five 450k datasets of varying ethnicity and cell types, and then empirically estimated significance thresholds both specific to the 450k array product and more generally for any EWAS study.

The results of the correlation analysis reveal a distinctive relationship, whereby proximal sites, up to a distance of around 1 kb apart, show a moderate level of correlation in the ∼0.25 to ∼0.4 range, falling to background levels once inter‐pair distances reach around 2 kb. These results are consistent with previous findings, which have found moderate to strong correlations of between 0.26 (Jaffe et al., 2012) and 0.45 (Ong & Holbrook, 2014) extending over genomic distances of between 1 and 2 kb (Eckhardt et al., 2006; Jaffe et al., 2012; Kuan & Chiang, 2012; Ong & Holbrook, 2014).

There do not appear to be any large‐scale differences in overall patterns of co‐methylation between brain and blood when comparing the Cau‐Am and Caucasian datasets, or between Gambian, Caucasian and African American populations when comparing the corresponding datasets. Only the CRC dataset shows some slight differences. Although it would be tempting to link this to the disease, such a result could perhaps equally arise from the smaller sample size, tissue heterogeneity or indeed data heterogeneity, especially given that all the datasets are taken from independent studies. Differences in the variability between sets are also apparent, with the spread of correlation values between adjacent bins of pairs showing a greater spread for the datasets having smaller sample sizes. Once again, this is merely speculated, as it is not possible to rule out data heterogeneity as the driver of such differences.

As far as we are aware, this has been the first attempt to characterise the overall patterns of co‐methylation between CpGs in the context of different tissues, ethnicities and disease states. The indications here are that there are unlikely to be any large‐scale differences in terms of the overall patterns of co‐methylation across tissues and ethnicities considered. There are however observable differences in co‐methylation patterns in the case of colorectal cancer. This is perhaps not all that surprising, given that large‐scale methylomic disruption is a hallmark of cancer (Sharma, Kelly, & Jones, 2010). However, before drawing any more general conclusions about the nature of this disruption and how it might relate to overall patterns of co‐methylation, further experimental work would be required. This could involve the collection of further samples and the generation of data under rigorously controlled conditions accounting for known sources of technical variation (e.g., batch effects) and confounding (such as age and smoking). Further, co‐methylation could be studied in a context‐specific manner, by categorising sites into CpG island, shores, shelves, and singletons.

We used a permutation scheme to generate a null distribution of test statistics and obtain values for α for each dataset. By using such a scheme, we were also able to circumvent issues concerning confounding due to technical and batch effects as well as cell type heterogeneity – usually important considerations in EWAS more generally. As an additional advantage, by electing to use only minimal statistical modelling, we are better able to demonstrate the robustness of the methods and maximise the potential applicability of the derived threshold. As for the results of the permutations, inspecting the fit of the beta distribution β(1, 467, 264) to the minimum *P* values generated, we see that the CRC dataset deviates from the expected distribution quite markedly. This was taken to indicate that this set did not produce reliable results, possibly due to its smaller sample size. For the Gambian, Caucasian, Afr‐Am‐GTP and Cau‐Am datasets, the beta distribution produced *P* values consistently inflated over the range, which suggests that an improved fit might be achieved by adjusting the second parameter of the beta distribution – in other words, changing the effective number of independent tests.

Looking now at the 5% values obtained, for the Gambian, Caucasian, Afr‐Am‐GTP and Cau‐Am sets, these are all larger than the Bonferroni adjusted 5% threshold of $\alpha =1.07\times 10^{-7}$. Assuming that the results and conclusions from the assessment of correlation within the different sets are more generally applicable, and that there are no major differences between different populations, then the results from these four sets can be combined to give a weighted mean of $\alpha =2.4\times 10^{-7}$. From permutation testing results, we conclude that a significance threshold of $\alpha =2.4\times 10^{-7}$ would be appropriate for the 450k, accounting for the subset of probes tested on the array but not the hypothetical set of probes that could be tested with saturated genome‐wide coverage.

To address the issue of genome‐wide multiplicity, we used a subsampling method to extrapolate the results of permutation testing to an array of infinite density. The results of fitting the Monod function to the subsampling data revealed that the limit for the number of tests as the coverage on the array becomes saturated is in the region of 1 × 10^6^, that is, 1 million CpGs, which is four times the density of the 450k array. The consensus of the results from the four different sets was taken, yielding genome‐wide $\alpha =3.6\times 10^{-8}$. Interestingly, this figure is close to that typically used for GWAS: $\alpha =5\times 10^{-8}$, although it is not clear why this would be the case, considering these are distinct molecular measurements. It is unlikely that this coincidence is due to pervasive effects of methylation quantitative trait loci, thus simply reflecting LD, because similar results were seen in Caucasian and Afr‐Am‐GTP populations. Further investigation is required to determine whether there is some common mechanism at play or that this is merely coincidence.

Comparing this with previous recommendations, we see that the estimate for a genome‐wide significance for EWAS obtained here is smaller than what has been suggested a liberal threshold of 10^−6^, and would fall within the range considered stringent, 10^−8^ to 10^−7^ (Rakyan et al., 2011). This threshold is however much less stringent than the genome‐wide Bonferroni level, which assuming there are 28 million CpGs across the genome is $\alpha =1.79\times 10^{-9}$. Bonferroni correction would then indeed be overly conservative for methylation data, but perhaps not to the extent previously suggested. Applying these thresholds to previous power studies for EWAS (Tsai & Bell, 2015), our results indicate that using the 450k‐specific $\alpha =2.4\times 10^{-7}$ would require sample sizes ∼10% larger than those previously estimated, and using the genome‐wide $\alpha =3.6\times 10^{-8}$ would require samples ∼20% larger to obtain the same power.

As for the limitations of our method, permutation correction attempts to identify a limit for the number of independent tests using a very small sample. The 450k offers only ∼2% coverage of the methylome, from which we have attempted to extrapolate a more general relationship between the 5% point of the minimum *P* values and the density of coverage. That our extrapolation reached a similar result for the four most reliable datasets we studied suggests that consistent information was present in the data. Although other models for extrapolation could be used, in GWAS data the Monod function was found to give similar results to extrapolation from full sequence data in limited regions (Pe'er, Yelensky, Altshuler, & Daly, 2008) as well as to population genetics simulations (Hoggart et al., 2008).

A related issue regarding extrapolation is whether the estimated genome‐wide threshold is more generally applicable in EWAS, for DNA methylation measurements generated from different tissues or populations, studying different diseases or utilising alternative array platforms or even technologies such as MeDIP‐seq (methylated DNA immunoprecipitation sequencing), WGBS (whole genome bisulphite‐sequencing) or RRBS (reduced representation bisulphite sequencing). In terms of cross‐tissue applicability, given the now wide‐spread availability of public 450k datasets, further work could be done in comparing thresholds derived for different tissues. In a similar vein, we could also repeat the study for different populations, or perhaps even perform a huge meta‐analysis using all healthy controls from every available study on GEO. Disease status is perhaps an important consideration, as wide‐scale disruption to the methylome such as that often observed in cancer could potentially invalidate some of the assumptions made, particularly regarding the level and extent of co‐methylation present, as the results we obtained here for the CRC data indicate. As for cross‐platform applicability, this is perhaps less certain, as arrays such as the 450k are designed to target genomic features such as promoter and enhancer regions and CpG sites in higher density contexts (Bibikova et al., 2009), resulting in relatively sparse and irregular genome‐wide coverage (Ong & Holbrook, 2014). In contrast to GWAS, where the design of SNP arrays is instead intended to maximise LD coverage across the genome, this could then mean that the results of extrapolation for EWAS are more reliant on the design parameters of the platform used, in terms of distribution of probes and regions covered (because co‐methylation structure is likely to vary according to genomic and functional context). The question of the extent to which the results depend on array design could be addressed in future studies using data from the latest platforms such as the EPIC array and comparing the results to those presented here. Ideally, simulation extrapolation would use data from a platform with even, un‐biased coverage of all features, which would increase confidence in the derived genome‐wide threshold. Such a threshold might then even find application in whole‐genome approaches such as WGBS, where although complete coverage of the methylome can be achieved, because permutation testing is computationally expensive, time consuming and study specific, an a priori estimate could still be desirable.

As experience in GWAS has shown, the use of a standard significance threshold aids comparison and combination of results across experiments, and enables power calculations to inform the design of future experiments. The $\alpha =2.4\times 10^{-7}$ threshold for the 450k array that we have estimated here takes into account both dependency between tests due to patterns of co‐methylation, and the multiplicity of the set of CpGs tested. As it is derived from results averaged over European, African and American populations, it could therefore find general application for 450k array data, offering an empirically derived, more permissive alternative to Bonferroni correction. One major limitation of this threshold is that it does not take into account genome‐wide multiplicity. The non‐random placement of probes and the current limited coverage of the 450k makes extrapolation to saturated probe coverage somewhat speculative, but we believe our estimated genome‐wide $\alpha =3.6\times 10^{-8}$ is a useful first step towards standardising levels of evidence in EWAS.

With the recent release of the EPIC array, the issue of significance in EWAS is as relevant as ever. Future work may then seek to apply the methods described here to similarly derive threshold estimates for the EPIC. In this regard, it is interesting to note that estimates for GWAS significance thresholds were based on early generation genotyping arrays, but remained unchallenged as denser arrays became available. We expect that comparison of future estimates of EWAS significance to the present results may go some way to addressing the question of whether it is possible to establish by extrapolation a universal, platform‐agnostic EWAS threshold for single site‐level differential methylation analysis.

## Supporting information

Supplementary Information

Supplementary Information

Supplementary Table 1. Permutation results for the additional small dataset consisting of samples from an African American population contained within GSE41826
Supplementary Figure 1. Details of the permutation algorithm
Supplementary Figure 2. Details of the subsampling algorithm
