# Supplementary material for: Estimation of a significance threshold for epigenome‐wide association studies
Source: Genet Epidemiol. 2017 Oct 15;42(1):20–33. doi: 10.1002/gepi.22086 (PMC5813244; doi:10.1002/gepi.22086)
Supplement: Supplementary file 3 — Supplementary Table 1. Permutation results for the additional small dataset consisting of samples from an African American population contained within GSE41826 Supplementary Figure 1. Details of the permutation algorithm Supplementary Figure 2. Details of the subsampling algorithm [file GEPI-42-20-s002.pdf]

Supplementary Table 1. Permutation results for the additional small dataset consisting of samples from an African American population contained within GSE41826

**Dataset**	n	α	m	b
Afr-Am	12	7.90E-08	633220	113038

Figure legends

Figure 1. Correlation versus genomic distance for pairs of probes in chromosome 1. a. Gambian, b. CRC, c. Caucasian, d. Afr-Am-GTP, e. Cau-Am

Figure 2. QQ plots showing observed distribution of minimum p values verses the expected distribution under complete independence. a. Gambian, b. CRC, c. Caucasian, d. Afr-Am-GTP, e. Cau-Am

Figure 3. Significance threshold as a function of CpG site density following the subsampling procedure. Where possible, the monod function was fitted to estimate an asymptote representing the threshold at fully saturated CpG density, this is indicated by a dashed red line. a. Gambian, b. CRC, c. Caucasian, d. Afr-Am-GTP, e. Cau-Am.

Figure 4. Estimated number of tests as a function of CpG site density. Where possible, the monod function was fitted, this fit is shown by the blue line. a. Gambian, b. CRC, c. Caucasian, d. Afr-Am-GTP, e. Cau-Am.

Supplementary Figure 1. Details of the permutation algorithm

Supplementary Figure 2. Details of the subsampling algorithm
